# The Search for Putative Hits in Combating Leishmaniasis: The Contributions of Natural Products Over the Last Decade

**DOI:** 10.1007/s13659-021-00311-2

**Published:** 2021-07-14

**Authors:** Patrick O. Sakyi, Richard K. Amewu, Robert N. O. A. Devine, Emahi Ismaila, Whelton A. Miller, Samuel K. Kwofie

**Affiliations:** 1 Department of Chemistry, School of Physical and Mathematical Sciences, College of Basic and Applied Sciences, University of Ghana, P. O. BOX LG 56, Legon, Accra, Ghana; 2 Department of Chemical Sciences, School of Sciences, University of Energy and Natural Resources, Box 214, Sunyani, Ghana; 3 Department of Medicine, Loyola University Medical Center, Maywood, IL 60153 USA; 4 Department of Molecular Pharmacology and Neuroscience, Loyola University Medical Center, Maywood, IL 60153 USA; 5 Department of Chemical and Biomolecular Engineering, School of Engineering and Applied Science, University of Pennsylvania, Philadelphia, PA 19104 USA; 6 Department of Biomedical Engineering, School of Engineering Sciences, College of Basic & Applied Sciences, University of Ghana, PMB LG 77, Legon, Accra, Ghana; 7 Department of Biochemistry, Cell and Molecular Biology, West African Centre for Cell Biology of Infectious Pathogens, College of Basic and Applied Sciences, University of Ghana, P.O. Box LG 54, Accra, Ghana

**Keywords:** Chemotherapeutics, Chemoinformatics, Natural products, Cytotoxicity, Leishmaniasis, Phenotypic screening

## Abstract

**Abstract:**

Despite advancements in the areas of omics and chemoinformatics, potent novel biotherapeutic molecules with new modes of actions are needed for leishmaniasis. The socioeconomic burden of leishmaniasis remains alarming in endemic regions. Currently, reports from existing endemic areas such as Nepal, Iran, Brazil, India, Sudan and Afghanistan, as well as newly affected countries such as Peru, Bolivia and Somalia indicate concerns of chemoresistance to the classical antimonial treatment. As a result, effective antileishmanial agents which are safe and affordable are urgently needed. Natural products from both flora and fauna have contributed immensely to chemotherapeutics and serve as vital sources of new chemical agents. This review focuses on a systematic cross-sectional view of all characterized anti-leishmanial compounds from natural sources over the last decade. Furthermore, IC_50_/EC_50_, cytotoxicity and suggested mechanisms of action of some of these natural products are provided. The natural product classification includes alkaloids, terpenes, terpenoids, and phenolics. The plethora of reported mechanisms involve calcium channel inhibition, immunomodulation and apoptosis. Making available enriched data pertaining to bioactivity and mechanisms of natural products complement current efforts geared towards unraveling potent leishmanicides of therapeutic relevance.

**Graphic Abstract:**

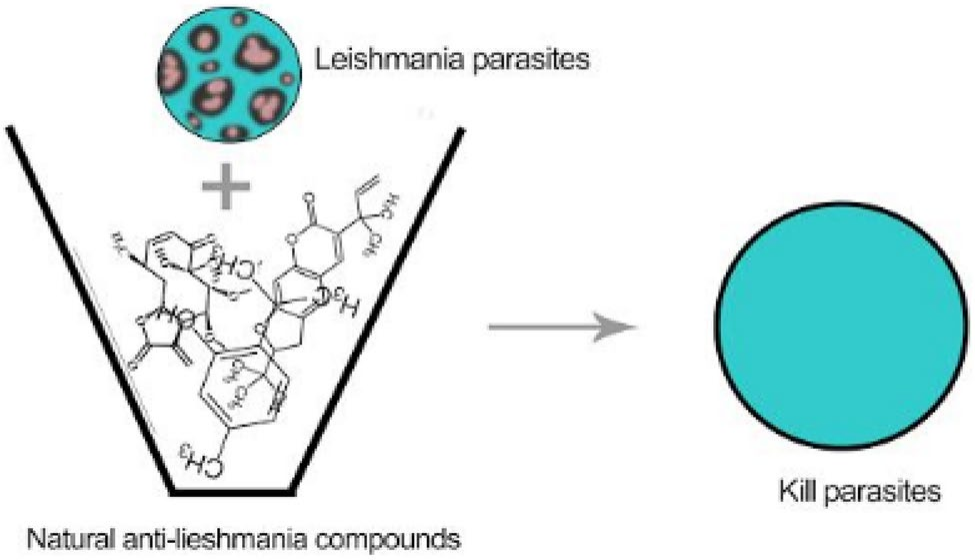

## Introduction

The debilitating rate of parasitic infections in the tropical and subtropical regions of developing countries has become alarming [1]. Vector-borne neglected tropical diseases and related synergetic co-infections, particularly leishmaniasis are very challenging and sophisticated to treat [2]. This is partly due to the existence of diverse parasitic species with different bionomics and sophisticated overlap between virulent factors. Activated immune response during disease exacerbation coupled with emerging resistance by both parasites and vectors against various treatment regimens have also contributed to this challenge [2, 3].

*Leishmania*, the etiological agent of leishmaniasis, is transmitted globally by over 90 different female sand-fly species of the Phlebotomus family, spread across 98 countries and four continents, with annual estimates of 1 million new cases and 30,000 deaths as at 2017 [2, 4]. The exact disease burden is unknown, but statistics indicate that over 350 million people are at risk, signifying a prominent public health risk [2, 5, 6].

Leishmaniasis is curable if the disease is diagnosed early and the appropriate medication is administered. Typically, leishmaniasis is initially marked by dermotropic ulcers, which then progress into the visceral tissues, resulting in a late and more debilitating condition that can often lead to death if left untreated. In some cases, the destruction of the mucocutaneous membrane especially the nose, throat, and mouth have also been very common [2]. The degree of clinical outcome and its corresponding immunopathology depends primarily on the type of causative species, age of host, and the balance between the host immune response and how the parasites subvert these defense mechanisms. In cases where the victim’s immune system is strong, *Leishmania* pathogens behave as opportunists by remaining dormant until the host’s immunity is compromised. Additionally, when the host is immunosuppressed, relapses are usually prevalent resulting in treatment failures.

Some challenges associated with the management of leishmaniasis include systemic toxicity of administrated drugs, high cost of existing therapeutic options, lengthy treatment periods and drug resistance. Furthermore, confounding factors such as parasite diversity has hampered various intervention strategies and halted global efforts, necessitating an immediate search for new drug leads for development as the next generation of antileishmanial agents [7–9]. In lieu of this, the review seeks to bring to the fore the various classes of natural products recently discovered with antileishmanial potentials over the last decade. Even though, the review primarily reported compounds with potent bioactivity, few with low potency were reported since these could be optimized or their scaffolds may serve as skeletons for the development of future leishmanicides.

### Trends in leishmanial chemotherapy and current panorama

Protection against leishmaniasis started with mimicking natural immunity through live inoculations [10] until modernized techniques including killed promastigotes and knocked out parasites came into play. Unfortunately, the presence of persistent lesions and the difficulty in estimating their efficacy rendered these approaches less effective [10, 11]. Efforts to alleviate leishmaniasis via chemotherapy include the use of pentavalent antimonial, which was essentially a small tartrate complex of antimony first reported in 1925 by Brahmachari [12, 13]. Although, antimoniate (Sb^V^) is still active after reduction by arsenate reductase to Sb^III^, *Leishmania* parasites are also susceptible to Sb^V^ via oxidative stress.

Gene amplification studies involving the Adenosine Triphosphate (ATP) binding cassette transporters including the multi-resistance proteins that act as efflux pumps have been shown to contribute to antimony resistance in clinical isolates [14, 15]. Likewise, deletions of aquaporin membrane carrier genes and phenotypic changes of the parasite with subsequent induced effects on the microbicide activity and the efflux rate of antimony reaching the macrophages also contribute to the resistance [16].

In the mid-1960’s pentamidine became the second choice to antimony resistant strains [17]. However, its utility like the antimonial was hampered due to severe vasomotor side effects and complex interactions with the pancreas which leads to the destruction of *β*-cells causing diabetes mellitus [17].

In the quest to expedite the time it takes for drugs to reach the market, strategies such as deciphering the cellular similarities between disease causing pathogens from phenotypic screening were developed. In the early 1960s, the anti-fungal amphotericin B from *Streptomyces nodosus* was used for treating leishmaniasis [18, 19]. This choice was widely accepted in most endemic areas due to its efficacy but not so in other areas especially East Africa (*L. donovani*) and South America (*L. infantum*) [20].

The anticancer agent alkyl phosphocholine (miltefosine) was the first oral formulation with strong protection against visceral leishmaniasis. Miltefosine works by modulating an apoptosis process induced by mitochondria membrane depolarization and phospholipid biosynthesis inhibition [21]. The main drawback in administering miltefosine for leishmaniasis treatment includes longer elimination time, lengthy treatment course, and miscarriage in pregnant patients after use [22].

A new and simple formulation of an old antibiotic paromomycin which inhibited translation with different modes of application (enteral, parenteral and topical) was also repurposed for leishmaniasis in 1967 [23, 24]. Unlike the other treatment options, paromomycin’s toxic effects are very minimal, but its efficacy is quite poor. New optimum carriers targeting pathogen macrophage using albumin has recently been reported to increase efficiency [25].

Following the failure of miltefosine, a collaboration between the Walter Reed Army Institute of Research (WRAIR, USA) and GlaxoSmithKline (UK) identified sitamaquine as a promising alternative, but its apparent loss of efficacy in tegumentary leishmaniasis limited its use [26]. Subsequently, findings from amphotericin B use and its high curative rate in patients influenced another repurposing strategy using the oral anti-fungal azoles (fluconazole, itraconazole, and ketoconazole) as suitable control and cost-effective therapy [27, 28].

Due to the therapeutic challenges, new chemotypes with high potency in tandem with immunostimulatory activity targeting new proteins applicable to both visceral and cutaneous leishmaniasis cases are desperately needed.

### Natural products as possible sources of new drugs against leishmaniasis

The lack of effective vaccines for control and concerted elimination campaign [2], and recent snail paced progress on leishmanial vaccine development does not guarantee any optimism. With the advancements in synthetic organic chemistry, combinatorial chemistry, and computational de novo drug discovery strategies, as well as high throughput screening techniques, only a few synthetically constructed drugs have been useful in combating leishmaniasis. Even with this, few natural product scaffolds represent major pharmacophores responsible for their curative effects. Between 2005 and 2010, about 19 natural products were registered for treatment of infectious diseases [29]. Similarly, over 69% of new small molecules used for the treatment of infectious diseases originated from natural products [30, 31].

Despite the large molecular weights of natural products which renders some of them less druglike, structural diversity, large chemical space and safety are characteristics that overrides synthetic alternatives. Treatments using extracts from plant families from endemic regions include *Fabaceae* [32], *Annonaceae*, *Euphorbiaceae* [31, 33, 34], *Rutaceae* [35–37], *Myrsinaceae* [31, 38], *Liliaceae* [39], *Araliaceae* [38], *Simaroubaceae* [40], as well as endophytes genera *Alternaria* [41]*, Arthrinium, Penicillium, Cochloibus*, *Fusarium, Colletotrichum, and Gibberella* [42]. Additionally, the exploration of marine natural products has led to the identification of interesting natural products with diverse biomolecular functions [43, 44].

Since the mid-eighties when the search for anti-leishmanial natural products became prominent, numerous metabolites originating from plants to current antileishmanial therapies have been reported. Lately, credible chemical entities from marine sources and endophytic species have also been reviewed [45–51]. This review presents the various classes of natural products from both flora and fauna that have been isolated over the last decade with anti-leishmanial properties. Also, the IC_50_/EC_50_ values and suggested mechanisms of action of these natural products are discussed.

### Classification of natural products with anti-leishmania properties

#### Alkaloids

Among the characterized bioactive constituents from nature, alkaloids have provided a broad-spectrum activity against different ailments and demonstrated their suitability as potential drug leads. Phenotypic alterations in ultrastructure form of the infective cells and immunomodulatory investigation studies of isolated alkaloids within the last decade reveal 27 alkaloids (Table 1) with varying efficacies from strong to weak activity. The natural product **3** isolated from *Cissampelos sympodialis* acts as a calcium channel inhibitor with immunomodulatory effects through the enhancement of nitric oxide (NO) production in macrophages [52]. Studies of **4** from *Croton pullei* reported significant alterations in organelle membranes of the endoplasmic reticulum, kinoplast and golgi body, depicting an apoptosis-like process [53]. Treatment with spectaline alkaloids, **16** and **17** from dichloromethane fractions of the flower *Senna spectabilis* of *Leishmania* promastigotes also portray a similar molecular mechanism like its structurally related piperine amide alkaloid, which either modulates the sterol biosynthetic pathway or acts as an inhibitor of cell proliferation by mitochondrion organelle destruction [54]. Although, the exact mode of action has not been fully elucidated, **21** from *Berberine vulgaris* like the active alkaloid in *Berberine aristate* perpetuates a similar activity through respiration incapacitation and apoptosis [55]. However, **21** was identified as a potential cell membrane disruptor via sterol biosynthesis inhibition [56], while **22** induces reactive oxygen species (ROS) generation. Structural activity relation (SAR) studies of high affinity protein kinase inhibitors, staurosporine-based compounds (**24-27**) revealed the 4th C methyl amine and 7th C hydrogen acceptor as the cause for the reinforced activity observed in *L. donovani*, which had major morphological changes in the flagella pocket and plasma membrane because of signal blockage via phosphokinase (PK) inhibition.

**Table 1:** 23 alkaloids isolated from various flora and fauna together with their IC_50_ and toxicity tested on some *Leishmania species*

Natural product source	Chemical structure	Class of natural product	IC_50_/μg/mL	Organism tested	Toxicology	References
*Paenibaccillus* sp. *(Marine)*		Imidazole	28.1	*L. donovani* (Promastigote)	Low toxicity profiles to mouse macrophages RAW 264.7 cell lines. > 250 µM	[57]
*Paenibaccillus* sp. *(Marine)*		Imidazole	0.203	*L. major* (Promastigote)	MIC = 25 μM	[58]
			1.90	*L. donovani* (Promastigote)		
*Cissampelos sympodialis*		Isoquinoline	80.0	*L. chasi* (Promastigote)	IC_50_ = 0.056 μM against human laryngeal cancer cells (HEP-2cells) and 0.067 μM against human mucoepide cells (NCIH-292)	[52]
*Croton pullei var. glabrior*		Piperidine	6.27	*L. amazonensis* (Amastigote)	Nontoxic as against murine macrophages after treatment with 79 µM of julocrotine	[53]
*Aconitum spicatum*		Pyrrolidine	56.0	*L. major*	No toxicity against MCF7, HeLa, PC3 cancer cell lines and 3T3 normal fibroblast cell line at 30 µM	[59]
			36.1			
*Helietta apiculata*		Quinoline	17.3	*L. donovani*		[60]
		Quinoline	25.5			
*Thalictrum alpinum*		Isoquinoline	0.175	*L. donovani*		[61]
			0.639			
			6.60			
*Trichosprum* sp.		Piperazine	96.3	*L. donovani*		[62]
		Piperazine	82.5	*L. donovani*		
*Piper choba*		Amide﻿	16.0	*L. donovani* (Promastigotes)	CC_50 =_ 0.76 μM and 0.83 μM against brine shrimp cells	[63]
		Amide	30.0			
*Senna spectabilis*		Piperidine	24.9	*L. major* (Promastigotes)	No observed lethality against J774 murine macrophage	[54]
*Aspidosperma ramiflorum*		Indole	18.5	*L. amazonensis* (Promastigotes)		[64]
			12.6			
*Beilschmiedia alloiophylla*		quinoline	2.95			[65]
*Berberis vulgaris*		Isoquinoline	2.10	*L. major* *L. tropica* (Promastigotes)	Observed toxicity against murine macrophage was at 9.18 μM	[66]
			2.90			
*Piper longum*		Amide	9.12	*L. donovani* (promastigotes)	Test against J774A.1 cell line indicated a high cytotoxicity at 5.05 ± 0.64 μg/mL. 393	[67]
			2.81	*L. donovani* (amastigotes)		
*Spongia* sp. *and Ircinia* sp. *(Marine)*		Indole	9.6		Toxicity profile against mammalian L6 cells was	[68]
Streptomyces sanyensis *(Marine)*		Indolocarbazole	0.0075	*L. amanzonensis* (promastigotes)	The series showed low selectivity against murine macrophage J774A.1 with CC_50_ of 5.20	[69]
			0.0012	*L. donovani* (promastigotes)		
			0.0002	*L. amanzonensis* (amastigotes)		
		Indolocarbazole	0.00017	*L. amanzonensis* (promastigotes)	8.74	
			0.0045	*L. donovani* (promastigotes)		
			0.0224	*L. amanzonensis* (amastigotes)		
		Indolocarbazole	0.037	*L. amanzonensis* (promastigotes)	> 40	
			> 0.089	*L. donovani* (promastigotes)		
			0.005	*L. amanzonensis* (amastigotes)		
		Indolocarbazole	0.0224	*L. amanzonensis* (promastigotes)	> 40	
			> 0.089	*L. donovani* (promastigotes)		

#### Phenolics

As characterized by hydroxy-phenyl groups, polyphenolics are widely distributed in nature and have been isolated from different plants. In traditional medicine phenolics have received much interest in phyto-therapeutics for the treatment of ailments ranging from non-infectious to infectious diseases. These chemotypes include compounds like coumarins, flavonoids, quinones, lignans, flavone glycosides amongst others (Table 2). Flavonoids from *Selaginella sellowi* when tested against different forms of *Leishmania* revealed a pro-drug mechanism for **28** but an activated NO generation for **29** [70]. The difference in the mode of action of these two flavonoids may be due to their conformational orientations. Similar investigations to understand the possible cause of apoptosis induced by **30** and **31** suggested a mitochondrial dysfunction with no influence on ROS [71]. However, evidence from suicidal action of some quercetin analogues have also indicated iron chelation, arginase inhibition, and topoisomerase II intercalation as possible mechanisms [72]. From the same *Nectandra* genus, inhibitory activity of **34** and **43** have been fully elucidated. Results indicated an inactivation of exacerbatory immunogens with reduced calcium levels and depolarized mitochondria potential [73]. Studies with similar compounds against melanoma cells indicated an apoptosis process confirming the depolarization activity [74]. Deciphering the exact mechanism underpinning the leishmanicidal action of isolated compounds from *Connarus seberosus*, it was revealed that defects in the mitochondria and plasma membrane structure with the evidence of lipid accumulation were caused by **55** and **56** [75]. Comparing **58** and its 3-*O*-methyl analog, **59,** to rosmarinic acid (based on the shared catechol nucleus), their potential mode of action is suggested as inhibition of reactive oxygen species [76, 77]. **75** as a chemo-preventive agent acts by reducing inflammatory symptoms by suppressing NF-κB expression and other pro-inflammatory factors including iNOS, COX-2, TNF-α, IL-1β, and IL-6 [78]. Compound **74** emulates an apoptosis induced suicidal mechanism which involves DNA fragmentation, inhibition of inflammation cytokines and the activation of caspases with downstream effects on gene transcriptional process [79]. Structural similarities of anti-inflammatory coumarins with **74** precludes a similar mechanism of action [80]. From the isolates of *Arrabidaea brachypoda* only **67** altered organelle structure and function by attenuating cytoplasm puncturing and golgi apparatus swellings [81].

**Table 2:** Various classes of phenolic compounds with their IC_50_ exhibiting antileishmanial properties

Natural product source	Chemical structure	Class of phenolic	IC_50_/μg/mL	Organism tested	Toxicology	References
*Selaginella sellowi*		Flavonoid	0.10	*L. amazonensis*	IC50 = 5.57 and 4.09 µM against Murine macrophages (J774.A1) and fibroblast cells (NIH/3T3)	[70]
		Flavonoid	2.80		CC50 = 5.75 and 47.4 µM against murine macrophage (J774.A1) and fibroblast cells (NIH/3T3).	
*Strychnos pseudoquina*		﻿Flavonoid	11.9 2.02	*L. infantum* *L. amazonensis*	Low-toxicity to infected murine macrophage up to 125 μM and low hemolytic activity in red blood cells	[71]
		Flavonoid	2.56	*L. amazonensis*	No significant toxicity > 199 μM	
*Lendenfeldia. dendyi* and *Sinularia dura* *(Marine)*		Phenyl ether	18.0	*L. donovani* (Promastigotes)	Low toxicity profile to VERO cells, pig kidney epithelia, human dermal carcinoma oral	[82]
		Phenyl ether	13.6	*L. donovani*	Ductile carcinoma breast, human malignant melanoma up to 13 µM	
*Nectandra leucantha*		Phenyl ether	8.70 6.00 34.9	*L. donovani* (Intra Amastigotes)	Nontoxic to mammalian peritoneal macrophages up to >293.8 μM 112.1 μM >292.1 μM	[83]
*Alpinia galanga*		Monolignols	10.5 16.6	*L. donovani* (Promastigotes)		[84]
		Phenol ester	8.80 5.60﻿			
*Hellieta apiculata*		Coumarin	35.8 27.5 32.1	*L. amazonensis* *L. infantum* *L. brazilensis*		[60]
		﻿Coumarin	18.5 27.4 21.5	*﻿L. amazonensis* *L. infantum* *L. brazilensis*		
*Nectandra oppositifolia*		Butanolide	3.58		Nontoxic against NCTC cell up to 42.3 µM	[85]
*P*iper regnellii *var.* pallescens		Lignan	5.00	*L. amazonensis*		[86]
*Nectandra cuspidata*		Flavonoid	38.5	*L. amazonensis* (Amastigotes)	Low cytotoxicity in J774.A1 macrophages	[87]
		Flavanoid	71.3			
		Flavanoid	34.0			
*Plumbago zeylanica*		Quinone	1.05 (EC_50_)	*L. donovani* (Amastigotes)	Very toxic to on RAW 264.7 macrophage cell lines	[88]
*Ocimum gratissimum*		Monolignol﻿	0.81	*L. infantum*	Nontoxic in murine macrophages RAW 264.7 cells lines 29.0 µM	[89–91]
		Monolignol	18.5		>100 µM﻿	
		Monolignol	14.9		97.7 µM	
*Vernonia polyanthes*		Quinone﻿	50.5	*L. amazonensis﻿* (Promastigotes)	At conc. > 52.4 µM in infected murine macrophages	[92]
		Quinone	10.2		At conc. > 37.23 µM in infected murine macrophages	
*Connarus Suberosus*		Chromanone	1.13 4.5 5.2	*L.amazonensis﻿* *(amastigotes)* *L. amazonensis* *(promastigotes)* *L.infantum (promastigotes)*	Toxic at 18.3 µM against murine macrophages.	[75]
		Lignan	11.4 15.5 7.1	*L. amazonensis* (promastigotes) *L.infantum (promastigotes)* *L. amazonensis* (promastigotes	Reduction cell viability was at 116 µM.	
*Piper aduncum*		Lignan	0.31 0.28	*L. amazonensis* (promastigotes) *L. braziliensis* (promastigotes)	Critical changes in the morphology of 3T3 fibroblast cell lines and its viability was observed at 25 µM and above.	[93]
*Hyptis pectinata*		﻿Flavonoid	2.5	*L. braziliensis* (promastigotes)	N.T	[76]
		Flavonoid	>36.0			
*Geosmithia langdonii*		Phenyl propene	0.05	*L. donovani* (promastigotes)	N.T	[94]
*Geosmithia langdonii*		Carbasugar Carbasugar	0.34 0.20	*L. donovani* (promastigotes)	N.T	[95]
*Ferula narthex*		Coumarin	43.77	*L. amanzonensis* (promastigotes)	N.T	[96]
		Coumarin	46.81			
		Coumarin	11.51			
		Coumarin	46.77			
*Arrabidaea brachypoda*		Flavonoid	0.004 0.017 0.013 0.024	*L. amanzonensis* (amastigotes) *L. amanzonensis* (promastigotes) *L. brazilensis* (promastigotes) *L. infantum* (promastigotes)	High lethality against macrophages at concentration above 20 μM	[81]
		Flavonoid	0.02 0.017 0.037 0.012	*L. amanzonensis* (amastigotes) *L. amanzonensis* (promastigotes) *L. brazilensis* (promastigotes) *L. infantum* (promastigotes)		
*Trixis antimenorrhoea*		Flavonoid	78 96	*L. amazonensis* *(*promastigote) *L. brazilensis* (promastigote)	N. T	[97]
		Flavonoid	19 5.8			
*Anogeissus leiocarpus*		Flavonoid	0.003	*L. donovani* (promastigotes)	CC_50_ > 100 µg/ml	[98]
*Sassafras albidum*		Lignan	15.8	*L. amazonensis* (Promastigote)	Nontoxic against BALB/c mouse macrophages up to 282	[36]
		Lignan	45.4		190	
*Zanthoxylum tingoassuiba*		Coumarin	57.7	*L. amazonensis* (Promastigote)	N. T	[99]
		Coumarin	70.0			
		Lignan	12.0			

#### Terpenes and terpenoids

Another group of secondary metabolites with interesting anti-parasitic activities are terpenes. Ultrastructural changes of **79** in phenotypic screenings indicated mitochondrial blebs and lipid deformities [100, 101]. **80** isolated from essential oils of *Tetradenia riparia* were found to distort promastigote structure especially the fate of its chromatin followed by an apoptosis process which is suspected to be caused by caspase activation [102, 103] (Tables 3 and 4).

**Table 3:** Various classes of terpenes and terpenoids with their IC_50_ exhibiting antileishmanial properties

Natural product source	Chemical structure	Class of natural product	IC_50_/μg/mL	Organism tested	Toxicology	References
*Parinari excelsa*		Triterpenoid	0.05	*L. donovani* (amastigotes)	Cell viability assay with L6 cell lines revealed the lethal concentration at 73.5 μg/mL	[120]
*Morinda lucida*		Monoterpenoid	1.17	*L. donovani* (promastigotes)		[121]
*Canistrocarpus cervicornis* *(Marine)*		Diterpene	4.00	*L. amazonensis* (Intra Amastigotes)	Non-toxic up to 515 µM in human macrophage strains J774G8	[100]
*Tetradenia riparia*		Sesquiterpene	2.45	*L. amazonensis* (Promastigotes)	high toxicity against mouse peritoneal macrophages = 1.69 µM	[122]
*Laurencia dendroidea* *(Marine)*		Sesquiterpene	10.8	*L. amazonensis* (Intra Amastigotes)	CC_50_ in macrophages and lymph nodes in amastigotes cervical BALB/c mice 160.2 and 172.8 µM	[123]
		Sesquiterpene	1.50		112.9 and 120.2 µM	
		Sesquiterpen	1.62		133.5 and 139.3 µM	
*Combretum leprotum*		Triterpene	3.30	*L. amazonensis* (Promastigotes)	Non-toxic against mouse peritoneal macrophages	[124]
		Triterpene	3.48			
		Triterpene	5.80			
*Vanillosmopsis arborea*		Sesquiterpene	10.7	*L. amazonensis* (Amastigotes)	Low cytotoxicity to macrophage J774.G8 cell lines 451 µM	[106]
*Croton cajucara*		Diterpene	20.0	*L. amazonensis* (Axenic Amastigotes)		[108]
		Diterpene	41.4			
		Triterpene	58.3			
*Croton sylvaticus*		Diterpenoid	10.0 10.0	*L. major* (Promastigotes) *L. donovani* (Promastigotes)	Observed toxicity was low at 247.83 µM	[125]
*Sterculia villosa*		Triterpenoid	15.0	*L. donovani* (Intracellular Amastigotes)	N.T	[126]
*Salvia deserta*		Diterpenoid	0.46	*L. donovani*	N. T	[35]
		Diterpenoid	3.30			
		Diterpenoid	7.40 29.4			
*Garcinia achachairu*		Monoterpenoid	10.4 18.4	*L. amazonensis* *L. brazilensis*	N. T	[127]
*Rapanea ferruginea*			24.1 6.10	*L. amazonensis* *L. brazilensis*	N. T	[127]
*Calea zacatechichi*		Sesquiterpene Lactone	1.89	*L. donovani*		[116]
		Sesquiterpene Lactone	0.771			
		Sesquiterpene Lactone	0.898			
		Sesquiterpene Lactone	1.74			
		Sesquiterpene Lactone	3.09			
		Sesquiterpene Lactone	1.60			
*Tanacetum parthenium*		Sesquiterpene Lactone	2.60	*L. amazonensis* (promastigotes)	Low toxicity towards J774G8 cells	[128]
*Plumeria bicolor*		Monoterpene lactone	0.409	*L. donovani* (Amastigotes)	CC_50_ = 20.6 µM	[129]
		Monoterpene Lactone	1.19		CC_50_ = 24 μM	
*Pseudelephantopus spicatus*		Sesquiterpene lactone	0.0794	*L. amazonensis*	High selectivity towards parasites as compared to mammalian cells with >100, > 100 µM and > 100 µM µM against Hela, L929 and B16F10 cell lines	[130]
		Sesquiterpene lactone	0.142		58.5 µM, > 100 µM and > 100 µM against Hela, L929 and B16F10 cell lines	
		Triterpenoid	0.451		Toxic towards RAW264.7, HONE-1, KB and HT 29 cell lines with 15.6 µM, 8.8 µM,8.2 µM and 4.7 µM respectively	
*Calea pinnatifida*		Sesquiterpene Lactone	1.73	*L. amazonensis* (Promastigotes)	At 4.11 µM, toxic to J774 macrophages	[115]
		Sesquiterpene lactone	4.24	*L. amazonensis* (Amastigotes)	75.5 µM	
*Spongia* sp. *and Ircinia* sp. *(Marine)*		Diterpene	0.75	*L. donovani*	Toxicity profile against mammalian L6 cells was 9.64	[68]
		Sesterterpene	5.60		127	
		Sesterterpene	4.80		83.1	
		Sesterterpene	10.2		> 217	
		Triterpene	15.9		>146	
		Sesterterpene	14.2		>254	
		Tetraterpene	18.9		4.36	
*Baccharis tola*		Diterpenoid	4.60	*L. brazilensis*	All compounds showed high cytotoxicity in human U937 macro phages with values lower than 347 μM	[131]
		Diterpenoids	5.30			
*Jatropha muitifida*		Diterpenoid	11.9	*L. donovani*	Low toxicity profile against VERO cells	[132]
		Diterpenoid	4.69			
		Diterpenoid	4.56			
*Psidium Guajava*		Triterpene	1.01	*L. infantum* (Axenic Amastigotes)	At conc. = 12.2 µM in mouse macrophage cell lines J774A.1	[133]
		Triterpene	1.32		At conc. = 20.8 µM against same cell lines	
*Cystoseira baccata* *(Marine)*		Diterpenoids	20.4	*L. infantum* (promastigotes)	Non-toxic up to 126.6	[134]
		Diterpenoids	44.5		84.5	
*Pseudelephantopus spiralis*		Sesquiterpene lactone	0.06 0.012	*L. infantum* (promastigotes) *L. infantum* (amastigotes)	1.47 ± 0.08	[135]
		Sesquiterpene lactone	0.02 0.005	*L. infantum* (promastigotes)	0.97 ± 0.07	
		Sesquiterpene lactone	0.244 0.048	*L. infantum* (promastigotes) *L. infantum* (amastigotes)	5.57 ± 1.9	
*Nectria pseudotrichia*		Sesquiterpene Lactone	0.092 0.023	*L. infantum* (promastigotes) *L. infantum* (amastigotes)	3.17 ± 1.0 Highly selective to parasites compared to VERO cells and THP-1 (a human leukaemia monocytic cell line). All > 200 µM.	[136]
		Sesquiterpenoid	0.063	*L. braziliensis* (amastigotes)		
		Monoterpene	0.104			
		Monoterpene	0.117			
		Monoterpene	0.37			
*Croton echioides*		Diterpenoid	0.11	*L.amansonensis* (promastigotes)	N.T	[137]
		Diterpenoid	0.027			
		Diterpenoid	0.025			
*Taxodium distichum*		Diterpenoid	2.5 0.52	*L. donovani* (promastigotes) *L. amazonensis*	High toxicity against HT-29 colorectal carcinoma cells	[138]
*Lippia sidoides*		Monoterpene	23.9	*L. amazonensis* (Promastigotes)	36.5 µM	[139]
		Monoterpene	11.0		>100 µM	
		Monoterpene	15.1		63.6 µM	
*Trixis antimenorrhoea*		Sesquiterpene	0.3 0.96	*L. amazonensis* *(*promastigote) *L. brazilensis* (promastigote)	N. T	[97]
*Bifurcaria* *bifurc*-*ata* *(Marine)*		Diterpene	18.8	*L.donovani*	Toxicity potential against L6 primary myoblast cell was observed at 56.6 µM	[140]
*Dictyota spiralis* *(Marine)*		Diterpene	15.47	*L. amazonensis* (promastigote)	23.4	[141]
		Diterpene	36.81	*L. amazonensis* (promastigote)	69	
*Stypopodium zonale* *(Marine)*		Diterpene	9	*L. amazonensis* *(amastigotes)*	8.4 μM	[142]
*Plumarella delicatissima* *(Marine)*		Diterpene	0.025	*L. donovani* *(amastigotes)*	Cytotoxicity potential against human lung carcinoma, cells exhibited low toxic potentials which were >50	
		Diterpene	0.026		>50	
		Diterpene	0.034		>50	
		Diterpene	0.022		>50	
		Diterpene	1.9		>50	
		Diterpene	4.4		>50	
*Laurencia viridis* *(Marine)*		Diterpene	8.36 28.26	*L. amazonensis* (Promastigote) *L. donovani* (promastigotes)	0.22	[143]
		Diterpene	7.00 18	*L. amazonensis* (Promastigote) *L. donovani* (promastigotes)	4.6	
		Diterpene	34.65		0.6	
		Diterpene	12.96		1.4	
		Diterpene	10.32		>100	
*Dysidea avara* *(Marine)*		Sesquiterpene	28.21 20.28 7.64	*L. infantum* (Promastigotes) *L. tropica* (Promastigotes) *L. infantum* (Amastigote)	Low toxicity against human microvascular endothelial cells and (human acute monocytic leukemia cells with CC50 62.19 and > 100 respectively.	[144]
		Sesquiterpene	7.42 7.08 3.19	*L. infantum* (Promastigotes) *L. tropica* (Promastigotes) *L. infantum* (Amastigote)	36.8 31.75

**Table 4:** Various classes of steroids, fatty alcohol, lignan, and butanolide with their IC_50_ exhibiting antileishmanial properties

Natural product source	Chemical structure		IC_50_	Organism tested	Toxicity	References
*Sassafras albidum*		Steroid	54.3	*L. amazonensis* (Promastigote)	Nontoxic against BALB/c mouse macrophages up to 182	[36]
		Fatty alcohol	19.9		157	
*Trametes versicolor*		Steroid	1.70	*L. amazonensis* (Amastigote)	Toxicity profile against peritoneal macrophages 42.9 μM	[152]
		Steroid	0.07		39.4 μM	
*Aspergillus terreus*		Steroid	11.2	*L. donovani*	N. T	[153]
		Steroid	15.3			
		Steroid	54.3			
		Butenolide	7.27			
*Solanum sisymbriifolium*		Steroid	6.60 3.10	*L. amazonensis* *L. brazilensis*	N.T	[127]
		Steroid	> 100 59.8	*L. amazonensis* *L. brazilensis*	N. T	
*Paecilomyces sp.* *(Marine)*			18.2	*L. amazonensis* (Intra-Amastigote)	Non-toxic up to 183 µM in mouse peritoneal macrophage.	[154]
			7.89	*L. amazonensis*		
*Musa paradisiaca*		Steroid	201	*L. infantum* (Amastigote)	Low toxicity profiles against mammalian raw cell lines 462 µM	[155]
		Steroid	185		569 µM	
		Steroid	127		1147 µM	
		Steroid	98.5		150 µM	
*Pentalinon andrieuxi*		Steroid	0.08 0.009	*L. mexicana* (promastigotes)		[150]
		Steroid	0.03 0.004	*L. mexicana* (amastigotes)		
		Steroid	0.06 0.009			
*Porophyllum ruderale*		Terthiophene	37 51	*L. amanzonensis* (amastigotes)	CC_50_ = 370 μg/mL CC_50_ = 335 μg/mL	[151]
Marine Cyanobacteria *(Marine)*		Macrolide	4.67 µM	*L. donovani* (amastigotes)	N.T	[156]

Halogenated terpenes **72** and **83** from *Laurencia dendroidea* which only differ primarily in a double bond character also targets the same organelle via redox perturbation [104, 105]. The natural product **87** from *Vanillosmopsis arborea* show promising activity through apoptosis induction characterized by mitochondrial dysfunction and oxidative stress [106]. Similar mode of action was reported for **87** isolated from *Tunisia chamomile* essential oil against *L. amazonensis* and *L. infantum* [107]. Effects of clerodone terpenes, **88**, **89** and **90** from the stem bark of *Croton cajucara* have been shown to obstruct ROS protection via trypanothione reductase inhibition [108].

Interest in marine natural products which led to the evaluation of marine terpenes like pentacyclic triterpene **92**, which exhibited an anti-inflammatory action with enhanced levels of T cells and Th1 cytokines when compared to its control [109].

Elucidation of the exact mechanism of action of four triterpenes from the roots of *Salvia deserta* showed that despite the strong antioxidant capacity of **93**, it also kill parasites by inhibiting isopentenyl diphosphate condensation with the major target being farnesyl diphosphate synthase [110]. Studies to also understand the molecular basics of **94** shows a similar action like **80**, but fragmentation of DNA strands has been described for diterpene **95** and **96** [111, 112]. Inhibition of oxidative pathways particularly IFN-*γ*-related signaling by similar diterpenoid quinones isolated from the roots of *Salvia officinalis* has also been shown to prevent disease proliferation and further protecting the host specie [113]. Recent studies in estimating the role of the energy production in the form of ATP in *Leishmania* with acyl phloroglucinol derivatives has revealed **97** as a mitochondria complex II/III inhibitor [114].

Like terpenes which are formed by the head to tail condensation of isoprene units, terpenoids (terpenes with oxygen-containing functional group) also represent a unique group of natural products with high functionalization and promising pharmacological activity. Isolation of six germacranolides from the leaves and stems of the *Calea species* have shown promising activities against *L. donovani* and *L. amazonensis* [115, 116]. Among them morphological assessment studies with **100** and **111** indicated alterations in the nucleus and mitochondria describing an apoptosis like process through the mitosis motor downregulation pathway [115]. Due to the similar core structure shared with germacra-1(10),11(13)-dien-12,6-olide a similar mechanism is envisaged for its counterpart **104** by aiding in generating ROS complementing the elucidated apoptosis process. The natural product **106** shares same structural core therefore may possess similar mode of action in addition to the inhibition of thiol-antioxidant enzymes [117]. Interestingly, **106** and its iso-conformer have also been disclosed to induce a pro-inflammatory inhibition via the NF-KB pathway [118]. On the other hand, **110** and **125** have also exerted multi-spectral activities including suppression of cell proliferation modulators and upregulation of microbicidal NO species [119].

#### Steroids

Steroids are a class of natural or synthetic organic compounds with three six membered rings fused with a five membered ring. Ergosterol, the main sterol in *Leishmania* parasite constitute a major component of the cell membrane and mitochondrion of the parasite which when inhibited leads to parasite death. **164** extracted from *Trametes versicolor* mimics *Leishmania* ergosterol due to similarities in core structure but a break in oxygen–oxygen bond in ergosterol peroxide unleashes oxidation on lipids, proteins and nucleic acids of the parasite by free radical reaction leading to serious toxicity to the *Leishmania parasite* [145]. Apart from the biological formation of bridge peroxides, the deleterious effects of other lanostane type steroids on membrane state and integrity causing parasite death has been reported [146, 147]. Also, anti-infective studies of *Sassafras albidum* and its bioactivity guided fractionation reported a sterol and fatty alcohol, **162** and **163** respectively [36] as promising antileishmanial compounds. **162** which differs from cholesterol at C24 position is believed to kill the parasite via an apoptosis mechanism involving DNA fragmentation, inhibition of inflammation cytokines and the activation of caspases [148, 149]. Evaluating the suicidal action of active isolates from *Pentalinon andrieuxii*, **181** induced changes in immune responses particularly via necrosis and apoptosis characterized by increase in IL2 and IFN-γ which insinuates the control of pro-inflammatory cytokines by anti-inflammatory counterparts [150]. **182** halted the process of electron transport and ATP generation in the mitochondria [151]. In addition, plasma membrane alterations with the administration of the other isolates depicts a sterol metabolism inhibition as a contributing factor to parasite death [151].

## Conclusion

Though humans and natural products did not co-evolve, chemical prototypes from natural origins have numerous targets in both human and animal diseases. Their structural diversity, large chemical space and safety are intriguing characteristics that makes them very attractive. Diverse biomolecular functions including anti-leishmanial potentials are possessed by various plant families including *Fabaceae, Annonaceae, Euphorbiaceae, Rutaceae, Myrsinaceae, Liliaceae, Araliaceae* and *Simaroubaceae*, as well as endophytes genera *Alternaria, Arthrinium, Penicillium, Cochloibus*, *Fusarium, Colletotrichum, and Gibberella*, and marine natural product possess.

Management of leishmaniasis is plagued with systemic toxicity, high cost of existing drugs, lengthy treatment periods, drug resistance and parasite diversity. Different classes of natural products such as alkaloids, terpenes, terpenoids, and phenolics are examples of compounds evaluated towards the treatment of leishmaniasis. They exert their antileishmanial activities through calcium channel inhibitors, immunomodulatory through the enhancement of NO in macrophages, alterations in organelle membranes of the endoplasmic reticulum, respiration incapacitation and apoptosis. Other antileishmanial related mechanisms include cell membrane disruption via sterol biosynthesis inhibition, reactive oxygen species (ROS) generation, iron chelation, arginase inhibition, topoisomerase II intercalation, suppressing NF-κB expression and other pro-inflammatory, and trypanothione reductase inhibition.
